# The heat shock response in plants: new insights into modes of perception, signaling, and the contribution of hormones

**DOI:** 10.1093/jxb/erae419

**Published:** 2024-10-16

**Authors:** Gönül Dündar, Veronica E Ramirez, Brigitte Poppenberger

**Affiliations:** Biotechnology of Horticultural Crops, TUM School of Life Sciences, Technical University of Munich, D-85354 Freising, Germany; Biotechnology of Horticultural Crops, TUM School of Life Sciences, Technical University of Munich, D-85354 Freising, Germany; Biotechnology of Horticultural Crops, TUM School of Life Sciences, Technical University of Munich, D-85354 Freising, Germany; USDA-ARS, USA

**Keywords:** Abscisic acid, brassinosteroids, heat shock factors, heat stress, nuclear bodies, phytohormones

## Abstract

Plants have evolved specific temperature preferences, and shifts above this range cause heat stress with detrimental effects such as physiological disruptions, metabolic imbalances, and growth arrest. To reduce damage, plants utilize the heat shock response (HSR), signaling cascades that activate heat shock factors (HSFs), transcription factors that control the heat stress-responsive transcriptome for activation of protective measures. While the core HSR is well studied, we still know relatively little about heat stress perception and signal integration or crosstalk with other pathways. In the last few years, however, significant progress has been made in this area, which is summarized here. It has emerged that the plant hormones brassinosteroids (BRs) and abscisic acid (ABA) contribute to heat stress tolerance by impacting the modes of activity of HSFs. Also, we began to understand that heat stress is sensed in different cellular compartments and that events in the nucleus, such as nuclear condensate formation via liquid–liquid phase separation, play a key role. In the future, it will be important to explore how these multilayered perception and signaling modes are utilized to understand how environmental context and developmental stage determine the outcome of heat stress effects on plant growth and development.

## Teaser

This review summarizes recent discoveries in heat shock sensing and responses in plants. Such discoveries have revealed that different cellular compartments participate and that hormones play a significant role.

## Introduction

Plants rely on an optimal temperature range for proper growth and development, and temperature increases can have significant effects. Heat stress restricts generative and reproductive growth, which substantially threatens yield stability and is of paramount relevance due to global warming (Lesk *et al.*, 2016; Zhao *et al.*, 2017).

**Fig 1.**
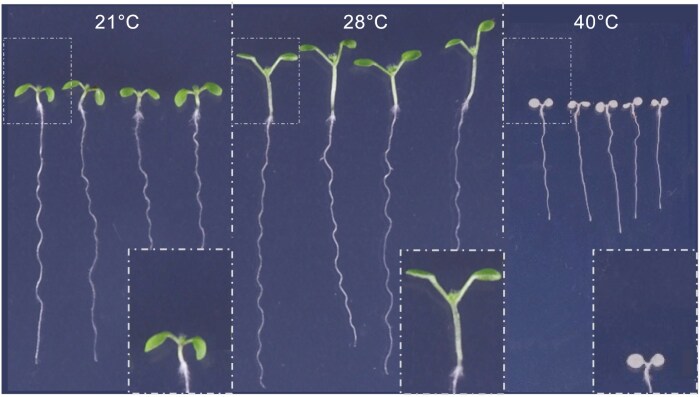
Effects of heat stress on the development of light-grown Arabidopsis seedlings. Seedlings grown for 4 d under long days at a temperature of 21, 28, or 40 °C differ in the elongation of roots, hypocotyls, and petioles, with 28 °C having promotive and 40 °C having repressive effects as compared with 21 °C.

Heat stress occurs when the ambient temperature surpasses a critical threshold for a duration sufficient to cause damage, and different species possess unique abilities to cope with heat stress. *Arabidopsis thaliana*, a plant from temperate climates, favors a daytime temperature of 20–25 °C for optimal vegetative growth, and while a rise of 5–10 °C (up to ~30 °C) can accelerate growth and certain developmental transitions, higher temperatures disrupt cellular processes such as photosynthesis, membrane structure, and protein integrity (Yeh *et al.*, 2012), and arrest growth (Fig. 1). To protect themselves, plants have evolved sophisticated heat acclimation techniques triggered by warmer priming temperatures. Notable reactions include growth adaptations such as increased elongation of stems and leaf petioles (Fig. 1), a process known as thermomorphogenesis (reviewed in Casal and Balasubramanian, 2019; Delker *et al.*, 2022), which confers cooling effects (Crawford *et al.*, 2012). In addition to morphological changes, diverse molecular and biochemical responses are triggered, all of which are conceived to increase the plants’ basal heat stress tolerance, enhancing their chances of survival in the event that heat stress ensues (reviewed in Zhao *et al.*, 2021).

A signaling route utilized for these protective measures is the heat shock response (HSR), a cellular stress response pathway conserved in all organisms and initiated not only by heat but also by other stress types that are proteotoxic, including exposure to certain toxins, heavy metals, or oxidative stresses (reviewed in Richter *et al.*, 2010). In such conditions, proteins, especially nascent proteins, can misfold or aggregate, resulting in their accumulation. To circumvent this, the HSR induces the expression of transcription factors called heat shock factors (HSFs) that bind heat shock elements (HSEs; nGAAnnTTCn) in the promoters of heat stress-responsive genes. An important target class is the heat shock proteins (HSPs), including the HSP70s and HSP90s. These molecular chaperones protect protein clients by buffering against misfolding and resolving aggregates (reviewed in Jacob *et al.*, 2017).

HSFs are present in all eukaryotes and have been extensively studied, particularly in mammals, where they bind HSEs in target promoters as trimers using a winged helix–turn–helix domain (Akerfelt *et al*., 2010). In Arabidopsis, there are 21 HSFs classified into A-type, B-type, and C-type, which contain the HSFA1–A9, B1–B4, and C1 groups (Scharf *et al.*, 2012). A-type HSFs are best studied, and among them are the HSFA1s (a, b, d, and e), which act as master regulators in the induction of the heat-responsive transcriptome (Yoshida *et al.*, 2011) by binding chromatin regions that become accessible after heat shock through structural reconfigurations (Huang *et al.*, 2023). In *A*. *thaliana*, the HSFA1s not only activate heat stress-responsive genes encoding HSPs, but also control other A-type HSFs, such as HSFA2, that together with HSFA1, or separately, allow for the maintenance of a sustained HSR and can confer a heat stress memory (Charng *et al.*, 2007; Friedrich *et al.*, 2021). HSFs of the B- and C-type lack the activation domain present in A-type HSFs and act in the repression of certain A-type HSFs, to maintain or re-establish an HSF equilibrium in both ambient temperature conditions and following heat stress recovery (Scharf *et al.*, 2012).

While the core signaling events of the HSR are well understood, we still know relatively little about how plants perceive elevated temperatures. However, significant progress has been made in the past few years, particularly in terms of elucidating events that occur in the nucleus and how hormones contribute (Box 1). This review focuses on the latest progress in this field.

Box 1. **Key developments in understanding heat stress perception**

Huang *et al.* (2023) described that heat stress induces rapid changes in chromatin architecture in tomato, which facilitates promoter–enhancer contact formation, a process that requires HSFA1a.
Albertos *et al.* (2022) showed that in Arabidopsis, the BR-induced transcription factor BES1 is de-phosphorylated by heat through the activity of ABA-repressed PP2C phosphatases, which enriches it in the nucleus and enables binding to HSEs in HSP promoters, a process facilitated by HSFA1s and BRs.
Luo *et al.* (2022) demonstrated that the BR-repressed GSK3 kinase BIN2 phosphorylates HSFA1d in Arabidopsis, to suppress its nuclear localization and inhibit its DNA binding activity, thereby allowing BRs to enhance heat stress resistance.
Tan *et al.* (2023) confirmed that HSFA1d is phosphorylated by BIN2 for cytoplasmatic retention. In addition, they show that HSFA1s, once in the nucleus, can interact with PIF4, which stabilizes PIF4 by interfering with PHYB interaction and promotes warmth-induced growth during the day for heat acclimation.
Bohn *et al.* (2024) identified the thermosensor TWA1, which changes its confirmation in response to heat stress for enrichment in subnuclear compartments via LLPS, where it interacts with TPL proteins and JAM transcription factors, and this promotes the expression of heat stress-responsive genes.

## Heat stress perception at the membrane and in the cytoplasm

Plants can sense and react to temperature fluctuations in different cellular compartments (Box 2), and a primary site is the cell membrane, where changes in fluidity, lipid composition, or membrane integrity occur and can be detected by membrane proteins (reviewed in Ding and Yang, 2022). Yet, while a membrane-bound cold stress receptor, the leucine-rich repeat receptor-like kinase PLANT PEPTIDE CONTAINING SULFATED TYROSINE1 RECEPTOR (PSY1R), was recently identified in *A. thaliana* (Peng *et al.*, 2024), the search for membrane-anchored heat stress receptors is still on, and future work will have to clarify if one even exists.

PSY1R mediates the cold-induced influx of Ca^2+^ ions through phosphorylation and activation of the Ca^2+^ transporter CYCLIC NUCLEOTIDE GATED CHANNEL 20 (CNGC20) and Ca^2+^ influx into cells; this process, however, is a primary response not only to low-temperature stress but also to high-temperature stress. It is known that heat induces an opening of Ca^2+^ channels, permitting an influx of Ca^2+^ ions from the apoplast; CNGCs implicated include CNGC2, CNGC4, and CNGC6 in *A. thaliana,* and CNGCb in the moss *Physcomitrella patens*, also required for the acquisition of heat stress tolerance (Finka *et al.*, 2012; Finka and Goloubinoff, 2014). In addition, the phospholipid-binding proteins ANNEXINs (ANNs) exhibit transmembrane transport activity; specifically, ANN1 and ANN4 contribute to a heat-induced increase of cytosolic Ca^2+^ concentrations (Qiao *et al.*, 2015; Wang *et al.*, 2015; Liao *et al.*, 2017). Increased Ca^2+^ levels are perceived by intracellular calcium sensors such as calmodulins, calcium-dependent protein kinases (CPDKs), and cyclic-dependent protein kinases, inducing the HSR via phosphorylation-mediated downstream events (reviewed in Ding *et al.*, 2020) (Box 2A). Ca^2+^ channels are activated by reactive oxygen species (ROS) and some, such as CNGC6, also account for elevated intracellular concentrations of nitric oxide (NO) in response to heat (Peng *et al.*, 2019; He *et al.*, 2022).

Heat stress detection also occurs in the cytoplasm, where heat-induced protein denaturation, misfolding, or aggregation is discerned. This can take place in the endoplasmic reticulum (ER), which initiates a secretory pathway called the unfolded protein response (UPR) that yields protein degradation, or in the cytosol, which triggers the HSR (Box 2B). Upon heat exposure, HSP abundance in the cytosol increases, and these proteins not only protect protein clients from misfolding but also function in heat stress signaling. The HSP70s and HSP90s bind HSFs, in particular HSFA1, in ambient temperatures to release them upon heat exposure for HSF enrichment in the nucleus and activation of the HSR (Box 2C). If HSP levels are in excess, for example, when the stress event has subsided, HSFs are bound, repressing the response for homeostatic regulation (reviewed in Jacob *et al.*, 2017; Ding and Yang, 2022).

## Heat stress responses in the nucleus

Once in the nucleus, HSFs can homo- or heterotrimerize, but they can also cooperate with other transcriptional regulators. One example is BRI1-EMS-SUPPRESSOR 1/BRASSINAZOLE-RESISTANT 2 (BES1/BZR2), a transcription factor controlled by the plant hormones brassinosteroids (BRs), which can interact with HSFA1a in the binding to HSEs for induction of the HSR (Albertos *et al.*, 2022). BRs are steroids that are best known for their growth-promoting capacities, which are conferred in a tissue-specific and environment-dependent manner by a phosphorylation-dependent signaling cascade. Upon BR perception by a receptor complex that contains the receptor-like kinase BRASSINOSTEROID INSENSITIVE 1 (BRI1), the subsequent interplay between the GSK3/shaggy-like kinase BR INSENSITIVE 2 (BIN2) and PP2A-type phosphatases such as BRI1 SUPPRESSOR 1 (BSU1) leads to the activation of BES1 and its homologs, including BZR1, through de-phosphorylation (reviewed in Kim *et al.*, 2009; Belkhadir and Jaillais, 2015).

BRs, when applied externally or when hyperaccumulated in plants, can reduce heat-induced damage, correlating with induction of HSP synthesis (Dhaubhadel *et al.*, 2002; Kagale *et al.*, 2007; Divi *et al.*, 2016; Sahni *et al.*, 2016). Interestingly, BR-promoted heat stress tolerance and HSP accumulation were not compromised in BR-deficient plants (Kagale *et al.*, 2007; Mazorra *et al.*, 2011), which had indicated that the response, although facilitated by BRs, does not rely on canonical BR signaling. In the past 2 years, significant progress has been made in understanding some of the underlying molecular modes. It was found that heat stress induced a strong de-phosphorylation and enrichment of BES1 in the nucleus (Ibañez *et al.*, 2018; Albertos *et al.*, 2022; Yao *et al.*, 2022), where it interacted with HSFA1a and promoted the expression of HSP70- and HSP90-encoding genes through HSE binding (Box 2C). In line with this, BES1 gain-of-function mutants exhibited increased heat stress tolerance, and BES1 loss-of-function mutants showed decreased tolerance, providing evidence that BES1 acts as a positive regulator of heat stress responses (Setsungnern *et al.*, 2020; Albertos *et al.*, 2022; Yao *et al.*, 2022). This function may be conserved among BZR-like proteins since a dominant mutation in BZR1, *bzr1-D*, also showed increased heat stress tolerance (Chen *et al.*, 2022).

Heat-induced BES1 de-phosphorylation has been shown to be independent of BRI1 since it was fully functional in the *bri1-1* mutant, which also failed to exhibit an altered heat stress tolerance (Albertos *et al.*, 2022). However, a different *bri1* knockout allele, *bri1-116*, showed decreased survival rates under heat stress (Luo *et al.*, 2022), thus indicating a potential multifactorial role for BRI1 in certain aspects of BR effects on the HSR, requiring further verification. Interestingly, BIN2, which phosphorylates and represses BES1 in BR signaling, was found to also target HSFA1d for phosphorylation to prevent its nuclear localization and activity in the HSR (Luo *et al.*, 2022; Tan *et al.*, 2023). Since both BIN2 and BES1 are clients of HSP90, which restrains their nuclear localization (Samakovli *et al*., 2014; Shigeta *et al*., 2015; Lachowiec et al., 2018), it seems possible that the HSR controls BES1 and BIN2 activities for feedback adjustment of heat stress signaling in the aftermath of the stress event.

Another factor that controls BES1 in the HSR is abscisic acid (ABA) since heat-induced BES1 dephosphorylation requires the ABA-repressed PP2C-type phosphatase ABA INSENSITIVE (ABI1) and its homologs (Albertos *et al.*, 2022). ABA is a sesquiterpenoid hormone that is biosynthesized in plastids and perceived in the cytoplasm by the soluble PYRABACTIN RESISTANCE/REGULATORY COMPONENTS OF ABA RECEPTOR (PYR/RCAR) proteins. This initiates complex formation with PP2Cs, inhibiting their activity in the dephosphorylation and repression of downstream targets such as SnRK2 kinases, leading to rapid phosphorylation and activation of downstream ABA-regulated transcription factors (Ma *et al.*, 2009; Park *et al.*, 2009). Interestingly, BIN2 is a protein target of ABI1 and its homolog ABI2, and BIN2 dephosphorylation by these PP2C-type phosphatases enables plants to activate BR responses in the absence of BRI1, enabling an interplay between the two hormones (Zhang *et al.*, 2009; Wang *et al.*, 2018).

ABA has a key role in abiotic stress tolerance, and it is well established that ABA synthesis strongly increases in response to different abiotic stress types, including drought, soil salinity, and osmotic stress (Singh and Roychoudhury, 2023). In heat stress responses, ABA appears to play a more complex role. While ABA levels do not significantly change after heat stress exposure (Prerostova *et al.*, 2020), both ABA signaling-deficient and ABA hyper-response mutants showed defects in basal and acquired thermotolerance (Larkindale *et al.*, 2005; Bohn *et al.*, 2024). ABA application and a loss of ABI1 function repressed heat-induced BES1 de-phosphorylation, which argues for an inhibitory role for ABA in certain stages of the HSR (Albertos *et al.*, 2022).

To better understand ABA activity in heat stress tolerance, a screen for mutants with altered ABA sensitivity under heat stress was performed, and this identified a novel nuclear thermosensor named THERMO-WITH-ABA-RESPONSE 1 (TWA1) (Bohn *et al.*, 2024). TWA1 contains a temperature-sensitive N-terminal domain which allows for its enrichment in subnuclear compartments in response to temperatures >30 °C, where it interacts with the transcriptional co-repressors TOPLESS (TPL), TPL-related (TPR), as well as JASMONATE-ASSOCIATED MYC-LIKE 2 (JAM2) (Box 2D). Nuclear body enrichment and TPL–JAM2 interaction were found to be required for the positive regulatory role of TWA1 in the induction of genes such as *HSFA2*, *HSP17.6*, and *HSP21* (Bohn *et al.*, 2024). Interestingly, TPL proteins also interact with BES1 (Ryu *et al.*, 2014; Espinosa-Ruiz *et al.*, 2017) and, since the BES1–HSFA1 interaction occurred in subnuclear domains (Albertos *et al.*, 2022), there is evidence that nuclear body formation is a more general, probably important feature of heat stress detection and signaling in plants.

## Nuclear condensate formation in heat stress sensing and signaling

Compartmentalization of macromolecules such as proteins and nucleic acids in subnuclear domains contributes to the formation of biomolecular condensates, which are membrane-less organelles that regulate different genetic and epigenetic processes, including chromatin modifications, transcription, mRNA splicing, and protein stability (Banani *et al.*, 2017; Shin and Brangwynne, 2017). Such condensates are formed via liquid–liquid phase separation (LLPS) to separate a strongly concentrated protein-rich phase from a dilute phase; proteins with intrinsically disordered regions or prion-like domains are often drivers of LLPS (Banani *et al.*, 2017). Furthermore, post-translational protein modifications such as SUMOylation, which is strongly increased by heat stress (Kurepa *et al.*, 2003), can facilitate the enrichment of proteins in subnuclear foci, adding another dimension to the dynamics of nuclear body assembly and disassembly (Saracco *et al*., 2007; Banani *et al.*, 2017).

In animal systems, one class of nuclear condensates includes the nuclear stress bodies, which are formed in response to heat shock and not only contain the HSFs HSF1 and HSF2 but have also been shown to function in processes such as transcriptional repression and mRNA degradation (Biamonti and Vourc’h, 2010; Morimoto and Boerkoel, 2013; Erhardt and Stoecklin, 2020). In plants, multiple lines of evidence suggest that the formation of nuclear condensates contributes to heat stress perception and signaling since not only HSFA1, BES1, and TWA1 can localize to subnuclear domains, but other factors potentially relevant for elevated temperature responses also can (Box 2D). This includes EARLY FLOWERING 3 (ELF3), a member of the evening complex that acts as a transcriptional co-repressor in flowering time control in *A. thaliana* (Nusinow *et al.*, 2011). ELF3 contains an unstructured prion-like domain that facilitates LLPS specifically in response to warmth, a transient process that promotes thermosensitive growth (Jung *et al.*, 2020; Hutin *et al.*, 2023) and may contribute to heat acclimation.

A class of nuclear condensates that have been very well studied are the designated photobodies, which contain the red/far-red light receptors PHYTOCHROMEs (PHYs) and are formed to regulate light-controlled growth (Chen *et al.*, 2010; Van Buskirk *et al.*, 2011). The PHYs interact with different proteins, including the basic helix–loop–helix transcription factors PHYTOCHROME INTERACTING FACTORs (PIFs) (reviewed in Cai and Huq, 2024). PIF4 and PIF7 control warmth-induced elongation growth through a promotive impact on BR and auxin biosynthesis and signaling, which is achieved, among others, through an interaction of PIF4 with BES1/BZR1 proteins (Sun *et al*., 2012; Ibañez *et al.*, 2018). Elevated temperature promotes PIF4 and PIF7 activity by multiple means (Kumar *et al.*, 2012; Chung *et al.*, 2020; Fiorucci *et al.*, 2020), including the stabilization of PIF4 through HSFA1 (Tan *et al.*, 2023) and disruption of photobodies that contain the red light receptor PHYB, a repressor of PIF4 activity that acts in temperature sensing (Jung *et al.*, 2016; Legris *et al.*, 2016). An intrinsically disordered region in PHYB, which promotes PHYB assembly in photobodies via LLPS, may also allow for temperature perception by yielding rearrangements in nuclear body composition depending on light and temperature context (Tan *et al.*, 2023).

In addition to phytochromes, other light receptors such as cryptochrome 1 and UV RESISTANCE LOCUS 8 (UVR8) contribute to temperature sensing by acting as negative regulators of PIF4 activity (Ma *et al.*, 2016; Hayes *et al.*, 2017). Interestingly, UVR8 can interact with BES1 to inhibit its DNA binding activity and repress BR-mediated growth (Liang *et al.*, 2018), potentially presenting another layer to the heat stress control of BES1. Indeed, an interesting question to ask is how warmth promotes growth through thermomorphogenesis whereas heat represses it. In this context, it is relevant to note that although the HSFA1s promoted BES1 activity in increasing *HSP* expression, they repressed BES1 activity in conferring growth-promoting capacities (Albertos *et al.*, 2022). Therefore, warmth may trigger the assembly of complexes that induce elongation growth, whereas heat stress may stimulate the formation of repressome complexes to repress BR-mediated growth responses and arrest growth (Box 2D). This may also allow for an integration of information and responsive decision-making, depending on the developmental and environmental circumstances that impact heat stress effects; it will be important to investigate these ideas in the future.

Box 2. **Summary of recent findings on heat stress sensing in different cellular compartments**

(A) Events at the membrane. In response to heat stress, the activity of Ca^2+^ channels such as CNGC2, CNGC4, and CNG6, as well as ANN1 and ANN4, increases, which yields Ca^2+^ ion influx and activation of Ca^2+^-responsive kinases such as CBK3, that activate HSFA1 via phosphorylation.

(B) Events in the cytoplasm. A heat-induced accumulation of unfolded or aggregated proteins is also detected, which triggers the UPR in the ER and promotes the HSR in the cytosol through the release of HSP-bound HSFs.

(C) Events in the nucleus. BRs can further promote the HSR via repression of BIN2 to release phosphorylation-conferred cytoplasmatic retention of BES1 and HSFA1d, resulting in their nuclear enrichment. ABA-repressed PP2C phosphatases also take part by promoting BES1 nuclear localization through de-phosphorylation for interaction with HSFA1s and induction of heat shock-induced genes such as HSPs.

(D) Events in nuclear condensates. Heat stress induces the formation of nuclear condensates, and one class contains TPL, TPR, JAM2, and TWA1. BES1, as a TPL-interacting protein, may also be contained in such repressome complexes and, via HSFA1 interaction, may repress growth and other processes that are inhibited by heat. UVR8, a BES1 interactor, may allow for an integration of UVR8-perceived stimuli into this response.

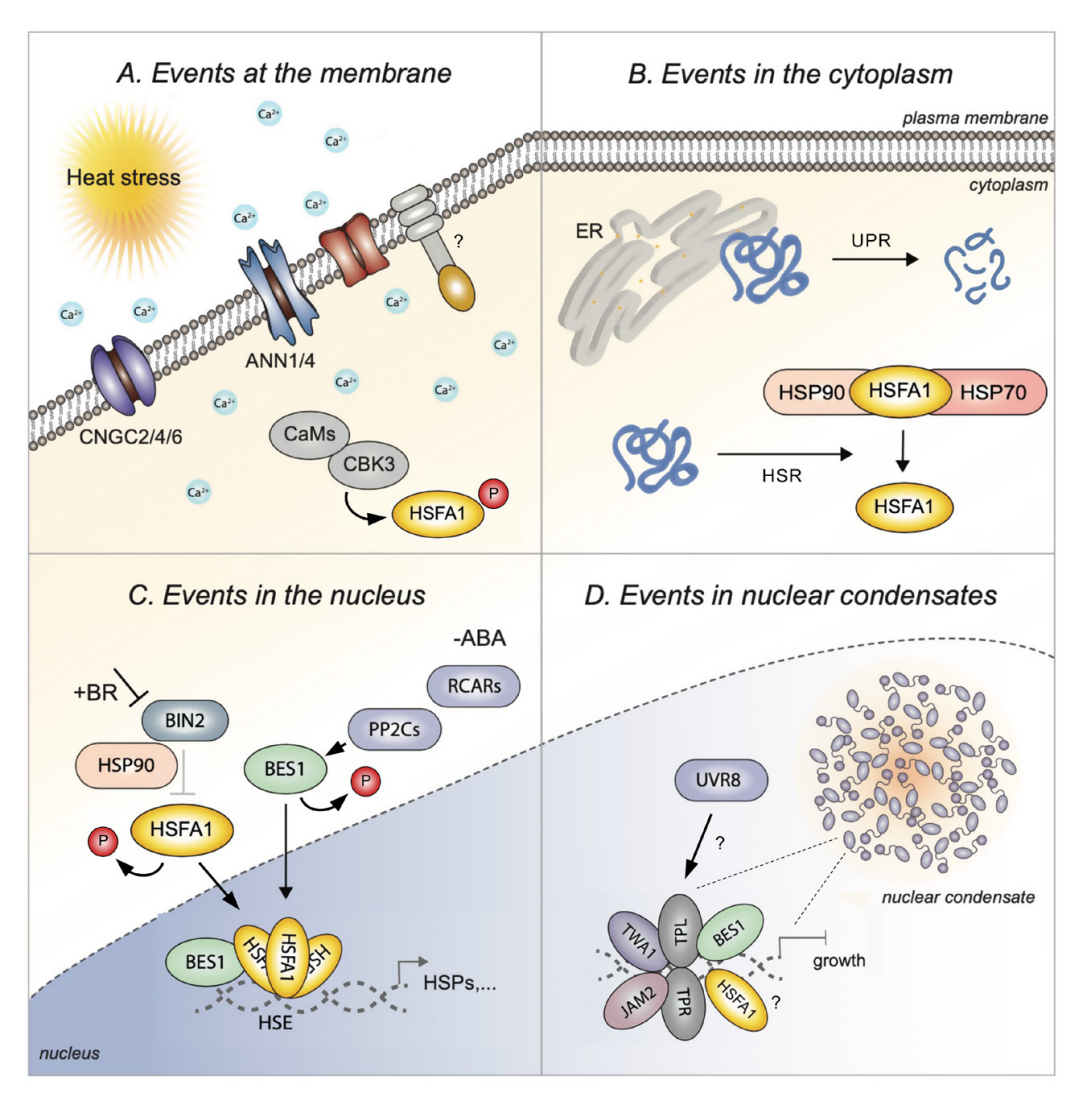

In summary, the highly dynamic process of nuclear condensate formation and disintegration may allow for a spatial enrichment of macromolecular complexes in specific chromatin regions in response to heat stress. The composition of these complexes probably determines whether gene expression is promoted or repressed, and may not be modulated exclusively by temperature. Additional environmental factors and developmental conditions may impact the degree of these heat stress-protective capacities.

## Conclusion

Heat stress is a consequential abiotic stress type that plants perceive and respond to in multiple cellular compartments, and further research is required to clarify if each perception mode is equally relevant. The canonical HSR utilizes the HSFs to activate the heat stress-responsive transcriptome for protective measures, including membrane structure changes, prevention of protein misfolding, and growth adaptation. In the past few years, we have learned to understand that HSF activities are diversified through interplay with components of other signaling cascades, including those that enable responses to light and hormones such as BRs and ABA. It will now be important to assess how such multilayered perception and signaling modes are utilized to integrate developmental and environmental contexts and determine the outcome of heat stress effects on plant growth and development.
